# *CDKN2A/p16INK4^a^* expression is associated with vascular progeria in chronic kidney disease

**DOI:** 10.18632/aging.101173

**Published:** 2017-02-09

**Authors:** Peter Stenvinkel, Karin Luttropp, Dagmara McGuinness, Anna Witasp, Abdul Rashid Qureshi, Annika Wernerson, Louise Nordfors, Martin Schalling, Jonaz Ripsweden, Lars Wennberg, Magnus Söderberg, Peter Bárány, Hannes Olauson, Paul G Shiels

**Affiliations:** ^1^ Division of Renal Medicine, Department of Clinical Science, Intervention and Technology, Karolinska Institutet, Stockholm, Sweden; ^2^ Department of Molecular Medicine and Surgery and Center for Molecular Medicine, Karolinska Institutet, Stockholm, Sweden; ^3^ Wolfson Wohl Translational Research Centre, Institute of Cancer Sciences, University of Glasgow, Glasgow, United Kingdom; ^4^ Division of Radiology, Department of Clinical Science, Intervention and Technology, Karolinska Institutet, Stockholm, Stockholm, Sweden; ^5^ Division of Transplantation Surgery, Department of Clinical Science, Intervention and Technology, Karolinska University Hospital, Stockholm, Sweden; ^6^ Pathology, Drug Safety and Metabolism, AstraZeneca, Mölndal, Sweden

**Keywords:** chronic kidney disease, vascular calcification, vascular senescence, vitamin K, p16

## Abstract

Patients with chronic kidney disease (CKD) display a progeric vascular phenotype linked to apoptosis, cellular senescence and osteogenic transformation. This has proven intractable to modelling appropriately in model organisms. We have therefore investigated this directly in man, using for the first time validated cellular biomarkers of ageing (*CDKN2A/p16^INK4a^*, SA-β-Gal) in arterial biopsies from 61 CKD patients undergoing living donor renal transplantation. We demonstrate that in the uremic milieu, increased arterial expression of *CDKN2A/p16^INK4a^* associated with vascular progeria in CKD, independently of chronological age. The arterial expression of *CDKN2A/p16^INK4a^* was significantly higher in patients with coronary calcification (p=0.01) and associated cardiovascular disease (CVD) (p=0.004). The correlation between *CDKN2A/p16^INK4a^* and media calcification was statistically significant (p=0.0003) after correction for chronological age. We further employed correlate expression of matrix Gla protein (*MGP*) and runt-related transcription factor 2 (*RUNX2*) as additional pathognomonic markers. Higher expression of *CDKN2A/p16^INK4a^*, *RUNX2* and *MGP* were observed in arteries with severe media calcification. The number of *p16^INK4a^* and SA-β-Gal positive cells was higher in biopsies with severe media calcification. A strong inverse correlation was observed between *CDKN2A/p16^INK4a^* expression and carboxylated osteocalcin levels. Thus, impaired vitamin K mediated carboxylation may contribute to premature vascular senescence.

## INTRODUCTION

When chronic kidney disease (CKD) progresses to end-stage renal disease (ESRD), the risk of cardiovascular mortality increases exponentially [1] and occur at a much younger age [2]. This suggests that CKD could be used as a clinical model to study premature vascular ageing [3, 4]. Premature arteriosclerosis, i.e. medial vascular calcification (VC), is a distinct feature in CKD that predicts poor outcome [5]. Osteogenic factors, such as matrix Gla protein (MGP) and runt-related transcription factor 2 (RUNX2), are part of the calcification process. MGP is an important endogenous inhibitor of extracellular calcification expressed by vascular smooth muscle cells (VSMCs) [6, 7], while *RUNX2* has emerged as a key factor in the osteogenic transformation of VSMC in response to high phosphate and oxidative stress [8, 9]. Uremic arteriosclerosis may in part be caused by increased vascular apoptosis [10, 11].

Studies have associated single nucleotide polymorphisms on chromosome 9p21 close to the cyclin-dependent kinase inhibitor 2A/B (*CDKN2A/B*) with risk for cardiovascular disease (CVD) in the general population [12-16]. As the proteins derived from *CDKN2A/B* are functionally involved in maintaining cells in a state of growth arrest, and cellular senescence increases with age, expression of *CDKN2A* increase as a function of increasing cellular stress and organismal ageing [17, 18]. Indeed, *CDKN2A* gene expression appears to be superior to telomere length as a biomarker of biological age [19, 20]. Thus, elevated *CDKN2A* expression correlates with increased biological ageing as well as increased frequency of a frail phenotype [21].

Since the expression of the CDKN2A/B locus has not been investigated in uremic tissue we investigated arterial and muscle *CDKN2A/B* expression in ESRD. We tested if the expression of the *CDKN2A/B* complex correlated to chronological age, degree of VC (estimated by histological examination of arteries, semi-automated quantification and CT scans of the coronary arteries) and clinical CVD. We also related degree of VC to the number of p16^INK4a^ and senescence associated β–galactosidase (SA–β–Gal) positive cells.

## RESULTS

**Figure 1:**
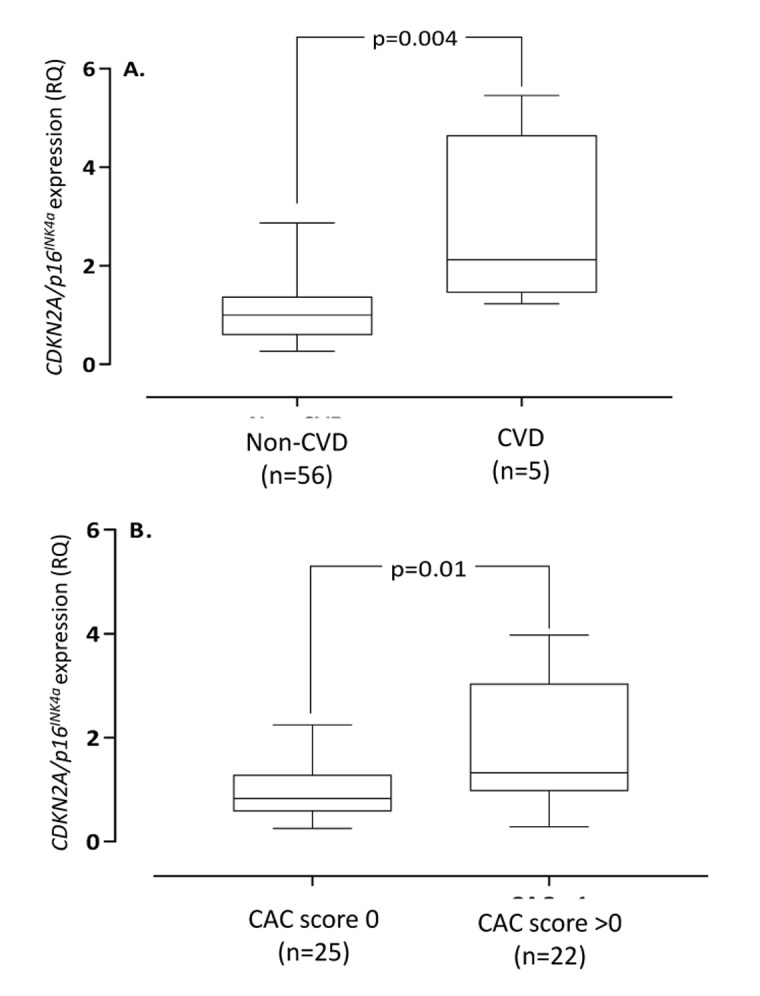
Arterial expression of *CDKN2A/p16^INK4a^* in end-stage renal disease patients with vs. without cardiovascular disease (**A**) and coronary artery calcification (CAC) score 0 vs. >0 (**B**). Cardiovascular disease was defined on clinical grounds. Coronary artery calcification score were obtained through CT heart (n=47). CDKN2A=cyclin-dependent kinase 2A. RQ=relative quantity.

Demography, biochemical data and arterial expression of *CDKN2A/p16^INK4a^*, *CDKN2B/p15^INK4a^*, *RUNX2* and *MGP* in the different groups of VC (none, mild, moderate and severe) are summarized in Table 1. No differences (p=0.15) in the number of no/former/present smokers were found in the calcification groups (no VC; 8/1/0, mild VC; 16/14/0, moderate VC; 7/7/0, severe VC; 3/1/0). Moreover, no difference (p=0.39) in physical activity (graded as regular, normal activity and disabled) was reported in the calcification groups (no VC; 2/6/1, mild VC; 18/13/1, moderate VC; 6/7/2, severe VC; 1/2/1). The arterial expression of *CDKN2B/p15^INK4a^* did not show any significant correlations with age, biomarkers of bone, inflammation and oxidative stress as well as extent of VC, coronary artery calcification (CAC) score or CVD. In contrast, the arterial *CDKN2A/p16^INK4a^* expression was significantly higher (Fig. 1) in patients with CVD compared to patients without CVD (median relative expression quantity 2.12 vs. 1.00, p=0.004), even after correction for age in multivariate analysis (p=0.01). Patients with CVD (632; IQR 79-1702) had significantly (p=0.0008) higher median CAC score than patients without clinical signs of CVD (0; IQR 0-64).

**Table 1:** Demographics, biochemical characteristics, arterial and muscle expression of the CDKN2A/B locus as well as the arterial expression of *RUNX2* and *Matrix Gla Protein*

**Demography**	**Total (n=61)**	**No VC (n=9)**	**Mild VC (n=32)**	**Moderate VC (n=16)**	**Severe VC (n=4)**	**P-value**
Age, years	45 (33-52)	28 (24-30)	44 (36-51)	49 (40-56)	53 (46-60)	**p<0.0001**
Males, n	42	5	20	13	4	p=0.1371 ^a^
Cardiovascular disease, n	5	0	1	2	2	**p=0.0464** ^a^
Vintage, years	0.3 (0-1.1)	0.7 (0-1.0)	0.3 (0-1.0)	0.6 (0-1.2)	1.4 (0.3-2.0)	p=0.8156
Body mass index, kg/m^2^	23.8 (21.8-26.3)	21.0 (19.8-25.7)	23.5 (21.7-26.2)	24.8 (22.6-26.7)	23.2 (22.3-26.8)	p=0.5453
**Metabolism**						
Creatinine, μmol/L	739 (577-907)	760 (519-1146)	761 (592- 986)	800 (598-906)	914 (806-933)	p=0.9366
Cholesterol, mmol/L	4.6 (3.9-5.3)	4.4 (3.9-4.8)	4.4 (3.8-5.1)	5.1 (4.3-6.0)	4.7 (3.8-7.2)	p=0.0853
Triglyceride, mmol/L	1.2 (0.9-1.7)	1.0 (0.7-1.5)	1.1 (0.9-1.5)	1.4 (1.1-1.8)	1.4 (1.1-2.9)	p=0.2950
HDL-cholesterol, mmol/L	1.4 (1.1-1.6)	1.6 (1.3-1.9)	1.4 (1.1-1.6)	1.3 (1.0-1.5)	1.2 (1.1-2.3)	p=0.4383
IGF-1, ng/ml ^b^	255 (175-289)	283 (261-326)	251 (183-291)	233 (175-282)	168 (147-370)	p=0.5116
Testosterone, mmol/L ^c^	12.3 (8.7-15.7)	11.2 (6.5-28.6)	13.1 (9.2-15.8)	11.0 (7.5-15.5)	10.7 (7.2-18.7)	p=0.6819
**Inflammation & oxidative stress**						
hsCRP, mg/L	0.9 (0.5-2.5)	0.6 (0.4-1.6)	1.3 (0.5-3.0)	0.8 (0.5-1.5)	2.0 (0.5-9.4)	p=0.8237
IL-6, pg/mL	0.9 (0.5-1.8)	1.0 (0.3-1.8)	0.9 (0.6-2.1)	0.6 (0.3-1.4)	1.7 (1.4-16.5)	**p=0.0480**
IL-8, pg/mL	5.3 (3.5-7.9)	5.9 (2.3-8.5)	5.4 (3.6-8.3)	4.4 (3.1-7.7)	7.4 (4.8-11.3)	p=0.4705
TNF, pg/mL	10.7 (9.0-13.1)	9.7 (8.0-14.7)	10.8 (9.3-12.9)	10.4 (8.5-12.6)	13.1 (12.2-16.7)	p=0.4624
Albumin, g/L	37 (33-39)	32 (31-38)	37 (35-39)	37 (33-39)	36 (32-42)	p=0.0754
Pentosidine, nmol/L ^d^	855 (609-1212)	650 (466-1735)	935 (691-1225)	752 (607-1393)	886 (696-984)	p=0.9495
8-OHdG, ng/mL ^d^	0.18 (0.13-0.27)	0.14 (0.08-0.19)	0.21 (0.15-0.26)	0.16 (0.10-0.27)	0.30 (0.04-0.42)	p=0.4616
**Calcification & bone markers**						
CAC score, HU ^e^	0 (0-111)	0 (0-0)	0 (0-90)	13 (1-171)	541 (28-1702)	**p=0.0154**
Media calcification, % ^f^	1.8 (0.5-5.5)	0.2 (0-0.3)	1.2 (0.5-2.5)	4.9 (0.3-9.7)	25.3 (2.5-32.9)	**p<0.0001**
Calcium, mmol/L	2.3 (2.2-2.4)	2.2 (2.1-2.4)	2.4 (2.2-2.5)	2.3 (2.2-2.4)	2.2 (2.0-2.3)	p=0.2136
Phosphate, mmol/L	1.8 (1.5-2.1)	1.6 (1.4-2.1)	1.8 (1.4-2.2)	1.9 (1.5-2.1)	1.4 (0.9-1.5)	p=0.2679
Magnesium, mmol/L	0.9 (0.8-1.0)	0.8 (0.7-1.0)	0.8 (0.8-0.9)	0.9 (0.7-1.0)	0.9 (0.7-1.1)	p=0.7395
iPTH, ng/L	258 (190-400)	216 (181-286)	252 (190-384)	371 (228-583)	127 (85-210)	p=0.1306
25-OH D-vitamin, mmol/L ^g^	39 (27-55)	33 (23-46)	37 (27-48)	51 (26-82)	39 (38-44)	p=0.1777
1,25-OH D-vitamin, pmol/L ^g^	18 (13-27)	17 (12-19)	18 (13-28)	18 (12-29)	18 (7-49)	p=0.6277
FGF-23, pg/ml ^d^	3353 (1114-31809)	5909 (1901-15150	3134 (453-33973)	4461 (1651-58853)	2184 (758-11994)	p=0.4184
Klotho, pg/ml ^h^	395 (297-520)	509 (434-625)	349 (260-480)	391 (307-489)	409 (320-499)	p=0.3608
Alkaline phosphatase, U/L ^i^	60.6 (49.6-89.2)	66.7 (47.2-126.1)	60.2 (50.3-82.7)	56.9 (47.9-78.1)	90.3 (47.0-113.1)	p=0.5853
Osteoprotegerin, pmol/L	5.9 (5.1-7.3)	5.3 (4.2-5.8)	6.1 (4.9-7.0)	5.8 (5.5-7.9)	9.3 (7.4-14.9)	**p=0.0049**
MID osteocalcin, ng/ml ^h^	83.6 (37.3-184.9)	76.4 (27.5-148.3)	125.4 (41.9-220.1)	80.6 (48.6-204.3)	24.9 (7.0-147.6)	p=0.4709
GLA osteocalcin, ng/ml ^j^	42.5 (22.3-66.0)	41.6 (26.7-62.4)	49.1 (22.4-66.8)	40.2 (27.8-74.7)	12.8 (8.5-27.3)	p=0.3225
GLU osteocalcin, ng/ml ^j^	28.4 (8.0-56.5)	25.5 (15.7-77.6)	33.2 (9.4-56.8)	31.3 (6.9-75.4)	2.6 (1.5-33.0)	p=0.5455
**Vascular expression (RQ)**						
*CDKN2B/p^15INK4a^* ^k^	1.35 (0.81-1.87)	1.91 (0.78-2.49)	1.28 (0.97-1.74)	1.58 (0.81-2.00)	1.16 (0.36-2.26)	p=0.3982
*CDKN2A/p16^INK4a^*	1.04 (0.25-1.02)	0.94 (0.45-1.20)	1.02 (0.62-1.62)	1.12 (0.49-1.69)	2.60 (1.18-5.05)	**p=0.0108**
*RUNX2* ^h^	1.56 (1.02-2.81)	2.09 (1.00-3.04)	1.12 (1.00-2.01)	1.55 (1.31-2.34)	6.12 (3.89-6.13)	**p=0.0036**
*Matrix Gla protein*^f^	0.88 (0.61-1.30)	0.70 (0.45-1.00)	0.87 (0.58-1.25)	0.91 (0.62-1.40)	2.33 (0.70-3.65)	**p=0.0002**
**Muscular expression (RQ)**						
*CDKN2A/^p16INK4a^* ^l^	0.63 (0.25-1.02)	0.35 (0.10-0.86)	0.45 (0.24-0.94)	0.63 (0.29-2.22)	0.88 (0.60-1.28)	p=0.7292

All continuous data are given as median (interquartile range).

HU = Hounsfield units; CDKN2 = Cyclin-dependent kinas; RQ = Relative quantity; PTH = Parathyroid hormone; hsCRP = high-sensitivity C-reactive protein; IL-6 = Interleukin-6; IL-8 = Interleukin-8; TNF = Tumour necrosis factor; HDL = High-density lipoprotein, 8-OHdG = 8-hydroxy-2′-deoxyguanosine, CAC = coronary artery calcification, RUNX2 = runt-related transcription factor 2 a; chi square, b; n=59; c; n=39, d; n=41, e; n=47, f; n=54, g; n=49, h; n=48, i;=44, j; n=52, k; n=58, l; n=50

**Figure 2:**
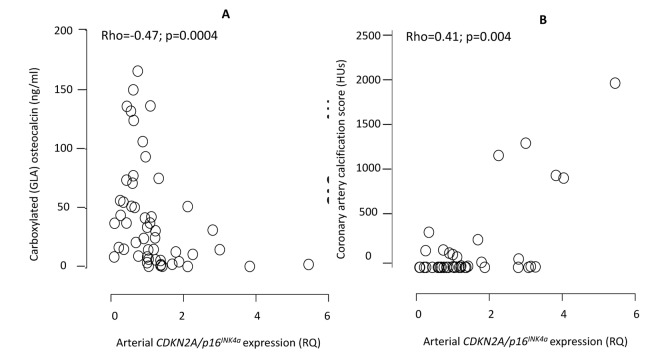
Correlations between the arterial expression of *CDKN2A/p16^INK4a^* and circulating levels of carboxylated (GLA) active osteocalcin (**A**) and coronary artery calcification by CT heart (**B**). CDKN2A = cyclin-dependent kinase 2A. RQ = relative quantity. HU = Hounsfield units. The exclusion of one patient on warfarin did not affect the correlation between *CDKN2A/p16^INK4a^* and GLA-OC (Rho=-0.51; p=0.001).

The arterial expression of *CDKN2A/p16^INK4a^* correlated (Rho=0.34; p=0.006) with chronological age (Suppl. Fig. 2). Patients with CAC-score >0 had significantly higher (median relative expression quantity 1.32 vs. 0.83 RQ; p=0.01) arterial expression of *CDKN2A/p16^INK4a^* than patients with CAC score 0 (Fig. 1), but not after correction for age (p=0.12). The correlation between *CDKN2A/p16^INK4a^* and media calcification was borderline (Rho=0.26; p=0.06) significant in univariate analysis (Suppl. Fig. 3) but reached statistical significance (p=0.0003) after multivariate correction for chronological age. The arterial expression of *CDKN2A/p16^INK4a^* correlated with CAC score (Rho=0.41; p=0.004), IL-8 (Rho=0.31; p=0.016), IGF-1 (Rho=-0.30; p=0.02), testosterone (males only) (Rho=-0.50; p=0.001), Mid-fragments (MID)-osteocalcin (OC) (Rho=-0.52; p<0.0001), inactive GLU-type (GLU)-OC (Rho=-0.54; p<0.0001) and active GLA-type (GLA-OC) (Rho=-0.47; p=0.0004). The correlations between *CDKN2A/p16^INK4a^* and GLA-OC and CAC score are depicted in Figure 2. Median N-MID OC (27.0 vs. 115.4 ng/ml; p=0.013), GLU-OC (3.1 vs 34.4 ng/ml; p=0.02) and GLA-OC (13.3 vs. 45.5 ng/ml; p=0.014) were significantly lower in patients with CVD.

**Figure 3:**
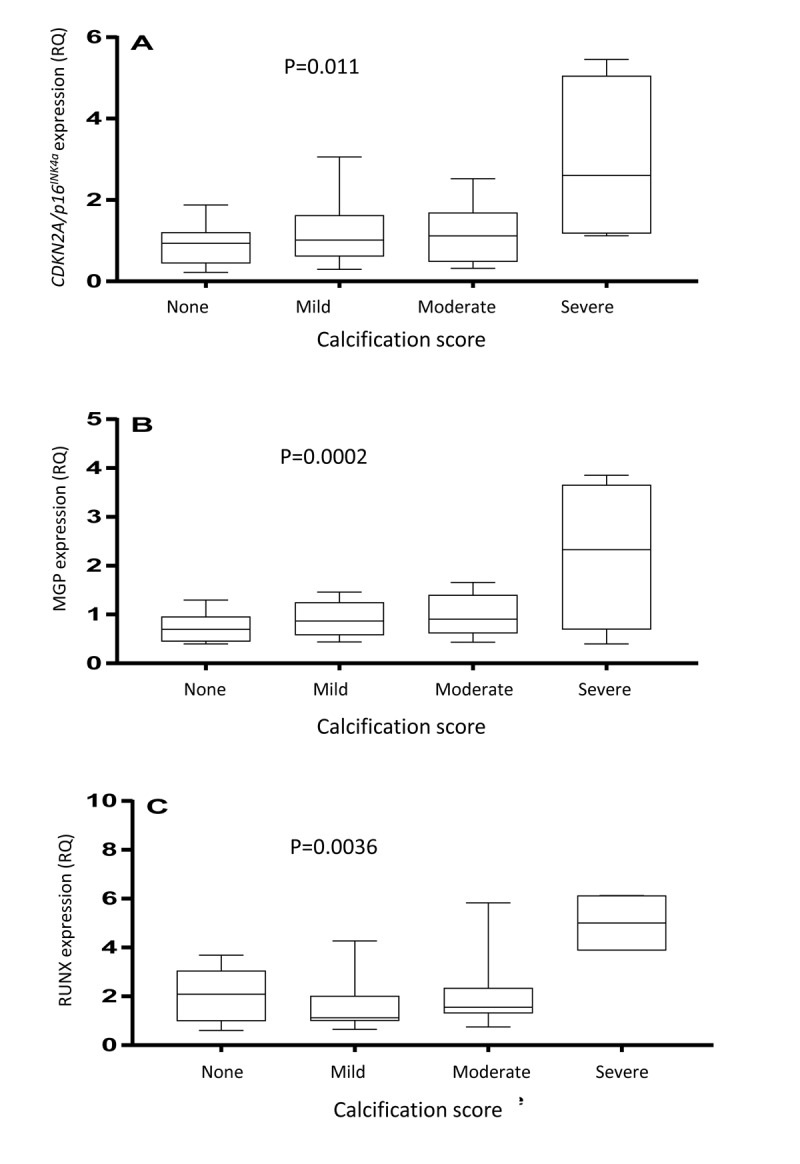
Arterial expression of *CDKN2A/p16^INK4a^* (**A**), matrix Gla protein (**B**) and runt-related transcription factor 2 (**C**) in arterial biopsies. The vascular biopsies from end-stage renal disease patients were stained with von Kossa and scored by a pathologist as having none, mild, moderate or severe media calcification. P-values represent ANOVA.

As shown in Figure 3, arteries with severe medial VC exhibited significantly higher vascular expression of *RUNX2* (p=0.0036), *MGP* (p=0.0002) and *CDKN2A/p16^INK4a^* (p=0.011). The arterial expression of *CDKN2A/p16^INK4a^* was significantly correlated to the expression of *MGP* (Rho=0.31; p=0.02), but not *RUNX2* (Rho=0.21; p=0.16). The degree of media calcification (%) correlated to total CAC score (Rho=0.59; p<0.0001) and *MGP* expression (Rho=0.38; p=0.008), but not to *RUNX2* expression (Rho=0.14). Total CAC score correlated to arterial *MGP* expression (Rho=0.38; p=0.016) but not to *RUNX2* expression (Rho=-0.01).

The expression of *CDKN2A/p16^INK4a^* did not differ between uremic arteries and skeletal muscle (median relative expression quantity 1.04 vs. 0.63 IU, p=0.79). No significant difference was observed in muscular *CDKN2A/p16^INK4a^* expression between patients with and without CVD (Table 1). The muscular expression of *CDKN2A/p16^INK4a^* correlated (Rho=0.30; p=0.02) with chronological age (Suppl. Fig. 2). A borderline significant (Rho=0.26; p=0.05) association was observed between the arterial and muscular expression of *CDKN2A/p16^INK4a^* (Suppl. Fig. 4). The muscular expression of *CDKN2A/p16^INK4a^* did not differ in patients with and without CVD nor between patients with CAC 0 vs. CAC >0 or the different calcification groups (Table 1).

**Figure 4:**
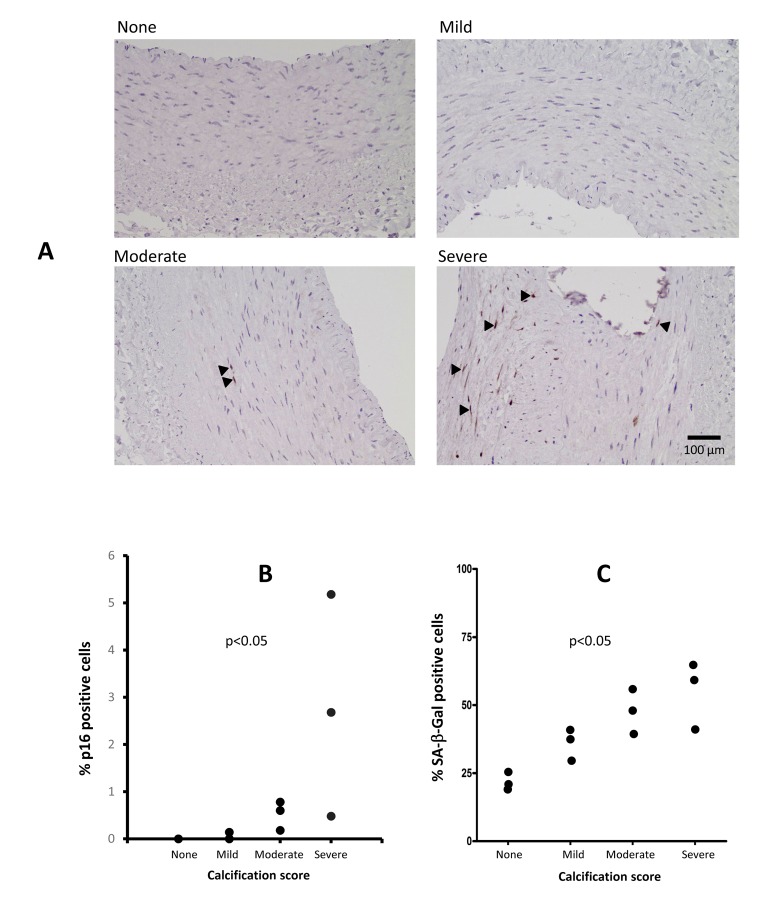
Immunostaining of arterial p16^INK4a^ and SA-β-Gal in patients with varying degrees of vascular calcification (VC) (**A**). The p16^INK4a^ expression was localised to the cell nucleus and involved more cells in severe VC. Staining for p16^INK4a^ with DAB (brown) and counterstaining with hematoxylin (blue). Arrowheads indicate p16^INK4a^ positive cells. (**B**) Levels of p16^INK4a^ in epigastric arteries from patients with varying degrees of VC, expressed as the percentage of p16^INK4a^ positive cells of total number of cells in the media and intima layers in a single arterial section (n=2 for no VC, n=3 for mild VC, n=3 for moderate VC and n=3 for severe VC). The number of positive SA-β-Gal positive cells increase with increased calcification (**C**); no VC; n=2, mild VC; n=3, moderate VC; n=3 and severe VC; n=3. P-values represent no vs. severe VC.

### p16^INK4a^ protein and SA-β-Gal expression

Expression of p16^INK4a^ in epigastric artery was absent, or low, in patients with no or mild VC, but was increased in moderate VC and further exacerbated in severe VC (Fig. 4). Cells positive for p16^INK4a^ were localized to the vascular media and in severe VC also in the intima and were more frequent (p<0.05) in areas with extensive calcification. The cellular localization of p16^INK4a^ was mostly nuclear. The p16^INK4a^ protein expression pattern mirrored the expression of *CDKN2A/p16^INK4a^*. The number of SA-β-Gal positive cells was significantly (p<0.05) higher in severely calcified arteries (Fig. 4).

## DISCUSSION

We report that the arterial *CDKN2A/p16^INK4a^* (but not *CDKN2B/*p15^INK4b^) expression associate with CVD as well as degree of VC in ESRD. These findings are corroborated by increased number of p16^INK4a^ and SA-β-Gal positive cells in severely calcified arteries (Fig. 4). Increased expression of p16^INK4a^ (and the cellular senescence that follows) may be causally involved in the decline of cellular function of stem and progenitor cells [22] that are observed with organismal ageing. A recent study in mice implies a significant role of *p16^INK4a^*/β-Gal^pH6^ positive macrophages and impaired immune system in aging [23]. Since vascular cell senescence contributes to breakdown of the blood-brain barrier [24] a consequence of increased vascular cell senescence may be higher cerebral concentrations of retained circulating cytokines, adipokines and uremic toxins. This may promote anorexia, cognitive dysfunction and depression; all common features in the uremic milieu [25, 26]. The senescence-associated secretory phenotype (SASP) - a key feature of cellular senescence - results in the secretion of pro-inflammatory factors that poison the tissue in the proximity of the senescent cell. In accordance, we report a significant correlation between circulating IL-8 and the arterial expression of *CDKN2A/p16^INK4a^* and higher IL-6 and OPG in severe VC (Table 1). Since senescent cells have been considered a target for anti-aging therapies and removal of p16^INK4a^-positive cells in progeric mice led to an attenuation of the ageing phenotype [27], a functional involvement of p16^INK4a^ in ageing-associated disorders is likely. The use of SASP modulators and senolytic drugs are intriguing prospect for the future treatment of uremic arteriosclerosis [28]. Indeed, a recent study in atherosclerotic mice show that chronic senolytic treatment improves established vascular dysfunction [29]. As we confirm that increased vascular senescence associates with low IGF-1 and testosterone levels [30, 31], the benefit of hormone replacement therapies needs consideration in this prematurely aged patient group.

This the first report on the expression of p16^INK4a^ in human uremic arterial tissue. Yamada et al. [32] showed increased arterial mRNA expression of *Cdkn2a* in rats with adenine-induced uraemia compared to control animals. Interestingly, they also demonstrated an increased arterial *Runx2* mRNA expression, and both *p16^INK4a^* and *Runx2* protein expression were detected in and around calcified areas of aortas from CKD rats, but not in normal rats [32]. We report higher arterial expression of *RUNX*2, *MGP* and *CDKN2A/p16^INK4a^* in ESRD patients with severe VC (Fig. 3). The higher *MGP* expression in arteries with severe media calcification may be regarded as an attempt to further prevent VC. Since VSMC apoptosis promote calcification [11], the relationship between the expression of the cell cycle inhibitor *CDKN2A/p16^INK4a^* and *MGP* is intriguing and requires further studies at the protein level.

The underlying cause(s) of the increase in *CDKN2A/p16^INK4a^* expression were beyond the scope of this study. Although it has been reported that oxidative stress may be a culprit [33] we find no associations between *CDKN2A/p16^INK4a^* expression and circulating oxidative stress biomarkers. Prelamin A accumulation promotes calcification and ageing in VSMCs – an effect which appears to be mediated, at least in part, by p16 protein. In addition, *RUNX2* may be activated by prelamin A and DNA damage signalling [34, 35], further linking cellular stress, p16 activity and an osteogenic potential. Another possibility for increased *p16^INK4a^* expression in ESRD is impairment of immune system function [23]. It was recently reported that the uremic toxin p-cresylsulphate induces macrophage activation, which lead to a failure in adaptive immune response [36]. Since we report on a strong inverse association between high vascular *CDKN2A/p16^INK4a^* expression and low levels of carboxylated active osteocalcin (Fig. 2) it can be hypothesized that vitamin K deficiency and decreased carboxylation of Gla proteins promote vascular senescence. Indeed, the expression of OC decreases with older age [37] and low carboxylation of OC associates not only with decreased cellular secretion of OC but also less accumulation in tissue [38]. Our data support Idelevich et al. [39] who report that OC, by promoting the osteocondrogenic differentiation in VSMC, is an active player in the VC process. Since vitamin K deficiency is a common finding in CKD [40] and promotes low carboxylation of OC our findings support a role for vitamin K deficit in premature vascular aging [41]. The protective effect of vitamin K may not only occur via carboxylation of Gla rich proteins but also via inhibition of apoptosis, which reduces the transdifferentiation of VSMC to osteoblasts [38]. It is noteworthy that vitamin K deficiency is consistently linked to features of aging, such as sarcopenia, frailty, osteoporosis and insulin resistance [42]. Since Vitamin K2 promote progenitor cell differentiation [43] its effect on vascular senescence, p16 clearance and progenitor cell mobilisation deserve further studies.

Some strengths and weaknesses of the present study deserve mentioning. An important strength is the access to highly biologically relevant methods and tissues. As opposed to being estimated by a surrogate marker of vascular stiffness the extent of VC was evaluated by three different methods. Moreover, gene expression data were obtained from the tissue directly affected by disease and we used von Kossa staining (the method of choice for detection of mineralisation). Finally, the presence of vascular senescence as estimated by arterial expression of *CDKN2A/p16^INK4a^* was mirrored by immunostaining of p16^INK4a^ and SA-β-Gal (Fig. 4). Weak points of the study include the cross-sectional design. As many of the findings are correlational in nature, conclusions on causation are impossible to infer and we cannot conclude on the pathogenic relationship between p16^INK4a^ overexpression and VC. However, since we observed a significant correlation between *CDKN2A/p16^INK4a^* expression and media calcification after correction for age our data imply that p16^INK4a^ is involved in the calcification process and not only a consequence of chronological aging. It should also be appreciated that patients in the study were a selected group of younger ESRD patients with a lower prevalence of CVD than the typical Caucasian dialysis patient (where a more aggravated vascular phenotype is expected). Thus, the results may not be applicable to older and sicker dialysis patients, nor dialysis patients of other ethnicities. Another limitation is the lack of data on vascular *CDKN2A/p16^INK4a^* and p16^INK4a^ expression in age- and gender-matched non-uremic patients with arteriosclerosis. Future studies would benefit from a control group, as it would make it possible to determine the impact of the uremic milieu on the degree of cellular senescence and *CDKN2A/p16^INK4a^* and p16^INK4a^ expression. It should also be pointed out that VC might vary depending on arterial type and location along any specific artery. However, we reported a close relation between epigastric artery medial calcification and extent of coronary artery calcification [44]. Finally, since RNA was prepared from a homogenised arterial sample, this precludes any knowledge on how the expression varied between different cell types. Still, the multiple associations of *CDKN2A/p16^INK4a^* with different markers of VC increase the strength of our findings.

In summary, we demonstrate that in the uremic milieu, increased arterial expression of *CDKN2A/p16^INK4a^* associated with vascular progeria, independently of chronological age. These findings are supported by the increased number of p16^INK4a^ and SA-β-Gal positive cell in arteries with severe VC. Further studies should evaluate if impaired Vitamin K mediated carboxylation contribute to premature vascular senescence.

## MATERIALS AND METHODS

### Patient population

Adult patients undergoing living donor kidney transplantation (RTx) at the Dept. of Transplantation Surgery at the Karolinska University Hospital were invited to participate in the study. All participants provided written informed consent and the Regional Ethical Review Board in Stockholm approved the study. Basic patient characteristics are outlined in Table 1. Whereas 39 patients had undergone dialysis treatment prior to RTx (median dialysis vintage 0.3 years) 22 patients underwent pre-emptive RTx. Eighteen of the individuals that were treated with dialysis had received haemodialysis (HD), 20 had received peritoneal dialysis (PD) and one had initially received PD but switched to HD. The median age was 45 years and 69% were males. Smoking status (no, former and current smoker) and degree of physical activity (high, moderate and low) were recorded. The most common causes of CKD were chronic glomerulonephritis (n=27), polycystic kidney disease (n=10), interstitial nephritis (n=3) and other (or unknown) causes (n=21). The median systolic and diastolic blood pressures were 142 (IQR 129-152) and 86 (IQR 74-93) mmHg, respectively. The most commonly used medications were erythropoietin-stimulating agents (84%), non-calcium-based phosphate binders (75%), calcium-based phosphate binders (4%) and loop-diuretics (62%). Commonly used anti-hypertensive treatments were β-blocking agents (52%) and ACE-i/ARBs (72%); 30% of the patients were on statins. One patient was on warfarin. In this group of younger patients only five had clinical signs of cerebrovascular, cardiovascular, and/or peripheral vascular disease (grouped as CVD). Three had clinical signs of ischemic heart disease and two had peripheral ischemic atherosclerotic vascular disease.

### Biopsy sampling

Within 20 minutes after skin incision, skeletal muscle and an artery (1-2 cm) were collected by sharp dissection. Muscle samples were taken from the external or internal oblique muscle or from the transverse abdominal muscle, and artery samples were obtained from the inferior epigastric artery. Samples were immediately placed in AllProtect Tissue Reagent (Qiagen, Hilden, Germany), snap-frozen in isopentane and subsequently stored at -70°C, or fixed in 4% phosphate buffered formalin. Formalin-fixed material from the epigastric arteries was embedded in paraffin. One or two separate tissue sections (1-2 μm thick) were stained with hematoxylin and eosin and van Kossa, respectively, and were evaluated by two experienced pathologists (AW and MS). The degree of medial calcification was semi-quantified on the van Kossa stained sections and graded 0-3, where 0 indicated no calcification and 3 the highest degree of calcification [44]. Degree of calcification was also assessed as a continuous variable by semi-automated quantification as previously described [44]. The correlation (p<0.0001) between semi-quantitative histological assessment and semi-automated quantification of media calcification is presented in Suppl. Fig 1.

### Biomarker measurements

Blood samples were obtained in a fasting state in the morning the day before the surgical procedure, and stored in -80°C. Interleukin (IL)-6, IL-8, TNF, IGF-1 and testosterone were analysed by immunometric assays on an Immulite 1000 Analyzer (Siemens Healthcare Diagnostics, Los Angeles, CA, USA) according to the instructions of the manufacturer. Plasma pentosidine was analysed by reverse-phase high performance liquid chromatography (HPLC). The following biomarkers were analysed with ELISA technique; 8-OHdG (Japan Institute for the Control of Aging, Shizuoka, Japan), osteoprotegerin (OGP) (Quidel Corporation, San Diego, CA, USA), human soluble α-Klotho (Immuno-Biological Laboratories Co., Ltd, Fujioka-shi, Japan), C-terminal FGF-23 (Immutopics Inc, San Clemente, CA, USA), Human N-MID Osteocalcin ELISA (IDS Nordic a/s, Herlev, Denmark), GLA-OC) and GLU-OC (Takara Bio Inc, Kusatsu, Japan). N-MID OC ELISA measuring total OC is more stable and reproducible than assays measuring intact OC only. Analyses of hsCRP, intact parathyroid hormone (iPTH), plasma cholesterol, triglycerides, HDL-cholesterol, creatinine, albumin, calcium, magnesium, phosphate, alkaline phosphatase (ALP), 25(OH) and 1,25(OH) D-vitamin were performed with validated routine methods at the accredited Clinical Chemical Laboratory Lab at the Karolinska University Hospital, Stockholm, Sweden.

### Computed tomography

All cardiac computed tomography (CT) scans were performed using a 64-channel detector scanner (LightSpeed VCT; General Electric (GE) Healthcare, Milwaukee, WI, USA) in cine mode. Scans were ECG-gated and a standard non-contrast protocol was used with a tube voltage of 100 kV, tube current of 200 mA, 350 ms rotation time, 2.5 mm slice thickness and displayed field of 25 cm. Calcium deposits in the coronary arteries were identified by a radiologist with Level 2 competence and extensive experience of cardiac CT interpretation. Data were subsequently processed and analysed using an Advantage Workstation (GE Healthcare). Smartscore 4.0 (GE Healthcare) was used to assess CAC scores. Calcified plaques were considered to be present if values exceeded the standard threshold of 130 Hounsfield units. The CAC scores were expressed in Hounsfield units (HU) as previously described in detail [45]. Total CAC score was defined as the sum of the CAC scores in the left main artery, the left anterior descending artery, the left circumflex artery and the right coronary artery.

### RNA preparation and gene expression analysis

TRIzol® Reagent (Ambion, Life Technologies, Carlsbad, USA) was used to prepare total RNA from artery and muscle biopsies according to the manufacturer's recommendation. RNA concentration was determined using a NanoDrop ND-1000 spectrophotometer (NanoDrop products, Wilmington, USA) and RNA integrity was evaluated by an Agilent 2100 BioAnalyzer (Agilent Technologies Inc., Santa Clara, USA). For further analysis only samples with RIN>5.5 were used. Reverse transcription reaction was performed using random hexamers and SuperScript^III®^ kit as per manufacturer's instructions (Invitrogen, Life Technologies, Carlsbad, USA). QPCR was performed using individual TaqMan® gene expression assays specific to *CDKN2A/p16^INK4a^* (#Hs00923894_m1), *CDKN2B/p15* (# Hs00793225_m1) and *HPRT1* (# Hs02800695_m1), as a reference gene, using 7500 Fast Real Time PCR (Applied Biosystems, Life Technologies, Carlsbad, USA). Gingell-Littlejohn et al. [20] previously published the primer sequences. Expression of *MGP(*Hs00969490_m1) and *RUNX2* (Hs00231692_m1) was analysed using a TaqMan Low Density Array on a QuantStudio^TM^ 7 Flex Real-Time PCR system (Life Technologies, Carlsbad, CA, USA) according to the manufacturer's instructions and 30-1000 ng of cDNA from epigastric artery was added to the array. Amplification profiles for each sample and target were analysed individually and profiles with Ct above 35, bad passive reference signal, noise spikes and high noise were removed from further analyses. The data were analysed using ExpressionSuite®Software v1.0.4 (Applied Biosystems, Life Technologies, Carlsbad, CA, USA), and the 2^-ΔΔCT^ formula was used to transform expression data from all genes to exponential values.

### p16^INK4a^ immunohistochemistry

Immunohistochemistry of p16^INK4a^ was performed on paraffin-embedded sections of epigastric arteries from 11 subjects. Samples were divided according to amount of VC; no VC (n=2), minor VC (n=3), moderate VC (n=3) and severe VC (n=3). Anti-*CDKN2A/p16*^*INK4a*^ polyclonal antibody was used (host species: rabbit, Antibody Registry ID: AB_306176, product number ab7962 Abcam, Cambridge, United Kingdom) and the analysis was performed using an established automated method at Dept. of Pathology, Karolinska University Hospital, Sweden. Staining and counterstaining were performed using diaminobenzidine (DAB) and hematoxylin, respectively. A section lacking primary antibody was used as a negative control. The score for p16^INK4a^ positivity was calculated as the percentage of p16^INK4a^ positive cells out of the total number of cells in the media and intima layers in one arterial section.

### Senescence associated β-galactosidase (SA-β-Gal) staining

SA-β-Gal staining was performed using Senescence Cell Histochemical Staining kit (Sigma-Aldrich, UK) with sections being fixed for 1 min in room temperature, washed three times in PBS and immersed in SA-β-Gal staining solution overnight in 37°C. Tissue sections were counterstained with eosin and viewed by bright-field microscopy. The percentage of senescent cells (SA-β-Gal positive cells) and the total number of cells was counted for each section in the six independent fields. Each group consisted of three tissue specimens.

### Statistical analysis

All continuous variables were expressed as median (interquartile range) and nominal variables as percentage, unless otherwise indicated. Statistical significance was set at the level of p<0.05. Comparisons between two groups were assessed with Wilcoxon rank sum test or χ^2^ test, as appropriate. Differences between the four groups were analysed by ANOVA. Spearman's rank correlation (ρ) was used to determine correlations between two continuous variables. Multivariate analysis was performed using general linear model (GLM). Statistical analyses were performed using statistical software SAS 9.4 (SAS Campus Drive, Cary, NC, USA 27513).

## SUPPLEMENTAL MATERIAL FIGURES


