# Supplementary material for: CDKN2A/p16INK4a expression is associated with vascular progeria in chronic kidney disease
Source: Aging (Albany NY). 2017 Feb 9;9(2):494–505. doi: 10.18632/aging.101173 (PMC5361677; doi:10.18632/aging.101173)
Supplement: Supplementary file 1 [file aging-09-494-s001.pdf]

SUPPLEMENTARY MATERIAL

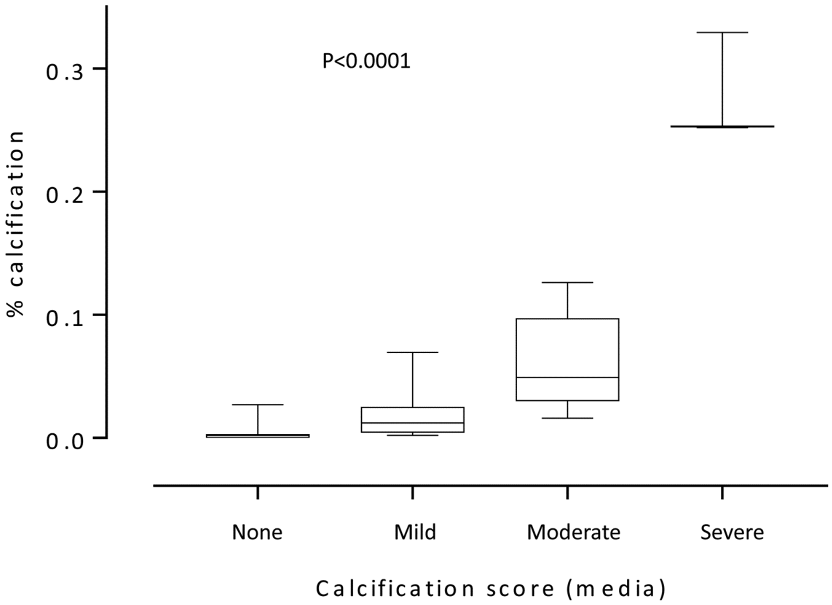

Suppl. Figure 1. The degree of calcification (%) as determined by semi-automated picture analysis in the four groups of calcification (determined by histological analysis by two pathologists).

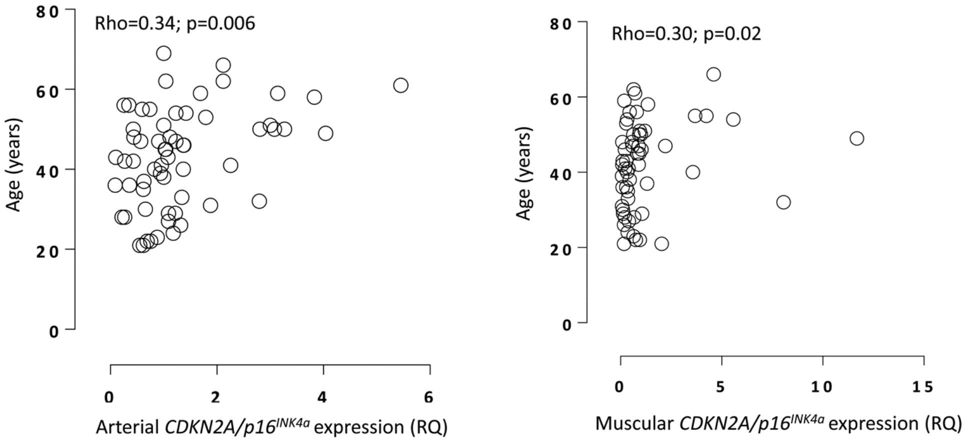

Suppl. Figure 2. Correlation between chronological age and the arterial (**A**) and muscular (**B**) expression of CDKN2A/p16INK4a.

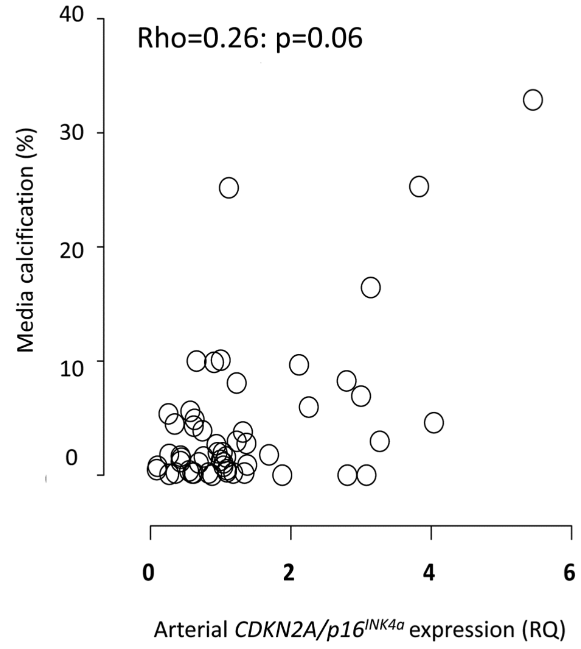

Suppl. Figure 3. Correlation between arterial expression levels of CDKN2A/p16INK4a and % calcified media (by semi-automated picture analysis). Following the correction for age the association achieved statistical significance (p=0.0003).

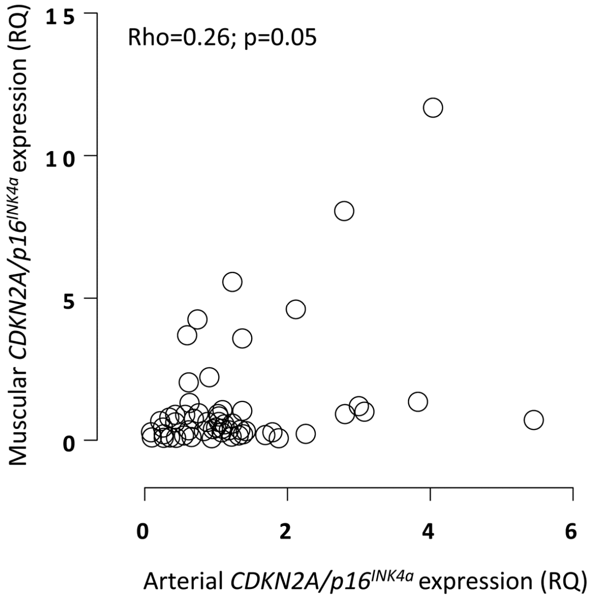

Suppl. Figure 4. Correlation between the arterial and muscular expression of CDKN2A/p16INK4a in end-stage renal disease.
